# An RNAi Screen Identifies New Genes Required for Normal Morphogenesis of Larval Chordotonal Organs

**DOI:** 10.1534/g3.118.200218

**Published:** 2018-04-20

**Authors:** Abeer Hassan, Yael Timerman, Rana Hamdan, Nitzan Sela, Adel Avetisyan, Naomi Halachmi, Adi Salzberg

**Affiliations:** Department of Genetics and Developmental Biology, The Rappaport Faculty of Medicine and Research Institute, Technion-Israel Institute of Technology, Haifa 3109601, Israel

**Keywords:** proprioception, chordotonal, morphogenesis, genetic screen, cell elongation

## Abstract

The proprioceptive chordotonal organs (ChO) of a fly larva respond to mechanical stimuli generated by muscle contractions and consequent deformations of the cuticle. The ability of the ChO to sense the relative displacement of its epidermal attachment sites likely depends on the correct mechanical properties of the accessory (cap and ligament) and attachment cells that connect the sensory unit (neuron and scolopale cell) to the cuticle. The genetic programs dictating the development of ChO cells with unique morphologies and mechanical properties are largely unknown. Here we describe an RNAi screen that focused on the ChO’s accessory and attachment cells and was performed in 2^nd^ instar larvae to allow for phenotypic analysis of ChOs that had already experienced mechanical stresses during larval growth. Nearly one thousand strains carrying RNAi constructs targeting more than 500 candidate genes were screened for their effects on ChO morphogenesis. The screen identified 31 candidate genes whose knockdown within the ChO lineage disrupted various aspects of cell fate determination, cell differentiation, cellular morphogenesis and cell-cell attachment. Most interestingly, one phenotypic group consisted of genes that affected the response of specific ChO cell types to developmental organ stretching, leading to abnormal pattern of cell elongation. The ‘cell elongation’ group included the transcription factors Delilah and Stripe, implicating them for the first time in regulating the response of ChO cells to developmental stretching forces. Other genes found to affect the pattern of ChO cell elongation, such as *αTub85E*, *β1Tub56D*, *Tbce*, *CCT8*, *mys*, *Rac1* and *shot*, represent putative effectors that link between cell-fate determinants and the realization of cell-specific mechanical properties.

The ability to sense the posture and movement of body parts based on signals from within the body is termed proprioception. In the fly larva, proprioception is mediated mainly by stretch-receptive chordotonal organs (ChO) (Caldwell *et al.* 2003) and specific subtypes of multiple dendritic neurons (Hughes and Thomas 2007; Song *et al.* 2007; Cheng *et al.* 2010). Eight ChOs develop in each abdominal hemisegment of the larva; five of them are clustered in the prominent lateral pentascolopidial organ (LCh5; Figure 1A). Each of the five scolopidia that constitute the LCh5 organ contains a bipolar neuron whose dendrite is ensheathed by a scolopale cell, and two accessory cells between which the scolopale cell is stretched: a cap cell at the dorsal side and a ligament cell at the ventral side. The cap and the ligament cells of the LCh5 organ are anchored to the cuticle by two cap-attachment (CA) cells (Ghysen and Dambly-Chaudiere 1989) and one ligament-attachment (LA) cell (Inbal *et al.* 2004), respectively (Figure 1B-C).

**Figure 1:**
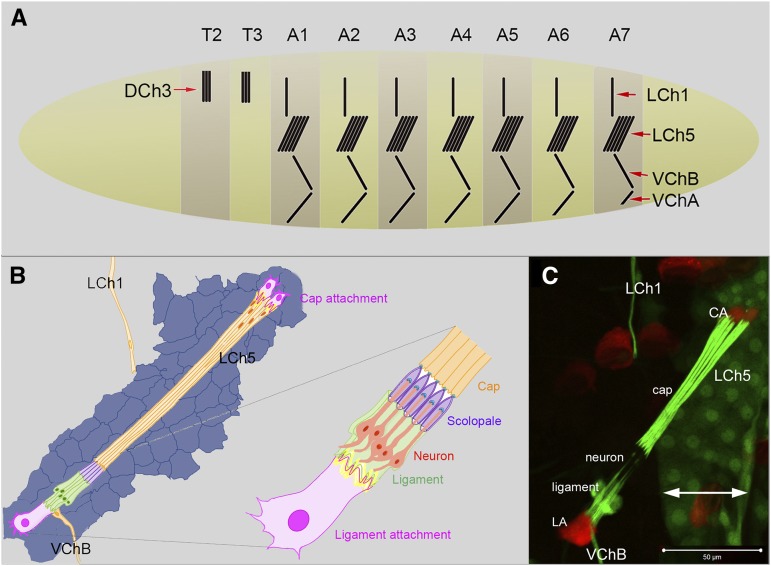
The larval chordotonal organs. (A) Schematic illustration of a first instar larva showing the eight ChOs (black bars) that form a zigzag line of stretch receptors in each of the seven abdominal segments A1-A7. Five ChOs are clustered in the pentascolopidial organ (LCh5). LCh1 is a single lateral ChO. VChA and VChB are two ventrally located ChOs. (B) Schematic illustration of a larval LCh5 organ. The organ is stretched diagonally from a dorsal posterior to a lateral anterior position in each abdominal segment between the epidermis (shown in blue) and the body wall muscles (not shown). The cap cells of the LCh1 and VChB organs are also presented. (C) An LCh5 organ of a second instar larva from the *en-gal4 UAS-GFP*, *dei^ChO^-GFP*, *dei^attachment^-RFP* reporter/driver strain used for screening. The cap and ligament cells express GFP (green) and the cap-attachment and ligament attachment cells express RFP (red). GFP expression is also evident in the epidermal stripe of En-positive cells (double-headed arrow). The scale bar = 50 μm.

The development of larval ChOs starts at mid-embryogenesis with the selection of ChO precursors from a cluster of *atonal*-expressing proneural cells (Jarman *et al.* 1993). Each precursor goes through several asymmetric cell divisions to generate the neuron, scolopale, cap, ligament and CA cells of a single organ (Brewster and Bodmer 1995). In parallel to the differentiation of the different cell types, which commences following the completion of cell divisions, patterning and localization of the organ as a whole take place. The LCh5 organ originates in the posterior dorsal region of each abdominal segment and it rotates and migrates ventrally to acquire its final position and orientation (Salzberg *et al.* 1994; Inbal *et al.*, 2003; Kraut and Zinn 2004). The ligament cells lead the migration process and pull the organ ventrally (Klein *et al.* 2010). Upon reaching their final destination the ligament cells recruit a LA cell through an EGFR-dependent mechanism (Inbal *et al.* 2004). During larval stages, with the dramatic increase in body size, the LCh5 organ, which remains anchored to the cuticle on both of its sides, elongates dramatically and goes through major morphological changes (Halachmi *et al.* 2016).

Whereas early steps in ChO development, namely the recruitment and specification of ChO precursors and the pattern of cell divisions, have been studied extensively (*e.g.*, (Jarman *et al.*, 1993; Lage *et al.* 1997; Okabe and Okano 1997; Brewster and Bodmer 1995), our knowledge about the genetic basis of later aspects of cell-fate determination, differentiation, morphogenesis and attachment of these organs is very sparse. To start filling in the large gaps in our knowledge about ChO development we have conducted an RNAi-based screen for new determinants of larval ChO organogenesis. Previous genetic screens for genes required for normal patterning of the embryonic peripheral nervous system (PNS) in general, or the ChOs in particular, were based on phenotypic analyses of the sensory neurons only (Salzberg *et al.* 1994; Kania *et al.*, 1995; Kolodziej *et al.*, 1995; Salzberg *et al.*, 1997). Thus, these screens could not identify genes that affect specifically the non-neuronal cell types (cap, ligament and attachment cells) or affect post-embryonic aspects of ChO development. There are two reasons for which screening in larvae, rather than in embryos, is critical for the identification of genes required for ChO morphogenesis: first, it has been recently shown that ChO morphogenesis is not completed during embryogenesis and that terminal differentiation and patterning takes place during larval stages (Halachmi *et al.* 2016). Thus, developmental defects that only become evident in larval stages are expected to be identified. The second reason is that only after hatching the ChOs start to experience significant mechanical stresses caused by larval growth and locomotion. Thus, genes required for the ability of the ChO to resist mechanical stresses and maintain organ integrity would not be identified by screening in the embryo.

Here we describe for the first time a screen that was performed on second instar larvae and focused on the accessory and attachment cells of the ChO, rather than the sensory neurons. The screen included 918 RNAi strains directed against 547 candidate genes. The genes were selected based on their expression pattern (enriched in ChOs), or potential function in cellular processes that seem critical for normal morphogenesis of ChOs, namely, tubulin-related genes and genes involved in cell migration. The screen identified multiple candidate genes required for different aspects of ChO morphogenesis, including the correct differentiation of specific cell types within the organ, proper attachment between the cap and CA cells and the normal pattern of cell elongation. The latter aspect of ChO development is especially interesting, as cell elongation in response to stretching forces probably depends, among other things, on the mechanical properties of the cell. Thus, the genes identified to be required for the normal pattern of cell elongation may provide a first insight into the formation of ChO cells with unique mechanical properties.

## Materials and methods

### Fly strains

Fly strains used in this study: *dei^ChO^-GFP*, *dei^attachment^-RFP* (Halachmi *et al.* 2016). The GFP-RFP marker chromosome was recombined to *en-gal4* (A. Brand, personal communication to FlyBase; Gramates *et al.* 2017), *ato-gal4* (Hassan *et al.* 2000) or *P{GMR12D06-gal4}* (Pfeiffer *et al.* 2008). For the analysis of *αTub85E* loss of function, we used the weak hypomorphic allele *Mi{PT-GFSTF.0}αTub85EMI08426* (Bloomington #60267). RNAi strains from the GD and KK libraries were obtained from the Vienna Drosophila Resource Center (VDRC); RNAi strains from the TRiP collection were obtained from the Bloomington Drosophila Stock Center, Indiana, USA. Two *dei* null alleles (*dei^KO-GFP^* and *dei^KO-mCherry^*) were generated as part of this study (GenetiVision, Houston TX, USA). First, the *dei^KO-GFP^* allele was generated by replacing the *dei* coding sequence spanning amino acid 23-366 with a MiMIC-like cassette (Venken *et al.* 2011), by injecting two gRNAs (GGCCAGAGCGACGGACTCCAAGG and GAATGGATACCCATCCAGAGCGG) and a donor plasmid, containing 3XP3 GFP flanked by *loxP* sites and inverted *attP* sites, into *nanos-Cas9* embryos (Bloomington #54591). The GFP-cassette was then replaced using Recombinase-Mediated Cassette Exchange (RMCE) with an *mCherry* cassette, using the plasmid *pBS-KS-attB1-2-GT-SA-mCherry-SV40* (obtained from the Drosophila Genomics Resource Center, IN, USA) for generating the *dei^KO-mCherry^* allele. The two *dei* null strains are fully viable. The *dei^KO-GFP^* strain expresses GFP mainly in the cap and ligament cells. The *dei^KO-mCherry^* strain expresses mCherry in a *dei*-like pattern.

### Collection and fixation of larvae

for 2^nd^ instar larvae, virgin females of the *dei^ChO^-GFP*, *dei^attachment^-RFP*; *ato-gal4*, or the *en-gal4*, *UAS-GFP*, *dei^ChO^-GFP*, *dei^attachment^-RFP* strain were crossed to males of the desired RNAi strain (∼30 females and 10 males). The flies were kept for 3-4 days at room temperature and then transferred to egg-laying chambers, put on grape juice plates with yeast paste and let to lay eggs for 24 hr at 29°. Adult flies were removed, and the progeny was left to mature at 29° for additional 20 hr. Larvae were washed once with phosphate buffered saline + 0.1% Tween-20 (PBT) and fixed overnight at 4° in 4% formaldehyde in PBT. Fixed larvae were washed twice with PBT (over 20 min) and twice with PBS (over 20 min) before mounting in Dako Fluorescent Mounting Medium (DakoCytomation, Glostrup, Denmark). Larvae were viewed using confocal microscopy (LSM 510, Zeiss) within a week from their fixation. Dissection and staining of 3^rd^ instar larvae were performed as previously described (Halachmi *et al.* 2012).

### Immunohistochemistry

Primary antibodies used in this study: Rabbit anti-Dei (1:50; Egoz-Matia *et al.* 2011), rabbit anti-αTub85E (1:50; Klein *et al.*, 2010) and mouse anti-αTub85E (1:5;(Nachman *et al.* 2015), mouse anti-Blistered/DSRF (1:00, a kind gift from S. Blair), MAb21A6 (1:20) was obtained from the Developmental Studies Hybridoma Bank, created by the NICHD of the NIH and maintained at the University of Iowa. Secondary antibodies for fluorescent staining were Cy3, or Alexa 647-conjugated anti-mouse or anti-rabbit antibodies (Jackson ImmunoResearch Laboratories, USA).

### Data availability

The strains generated in this work are available upon request. The authors affirm that all data necessary for confirming the conclusions of the article are present in the article, figures, and tables. File S1 contains the list of all RNAi constructs tested in this study. Table S2 lists the off-targeting effects of all tubulin-specific RNAi constructs used in the study. Supplemental material available at Figshare: https://doi.org/10.25387/g3.6165761.

## Results

### Genetic screen

Dissection and staining of large numbers of larvae is a slow and labor-intensive process. To overcome this limitation, we took advantage of recently developed ChO-specific fluorescent reporters that allow rapid screening of whole-mount larvae without any need for dissection or immuno-staining (Halachmi *et al.* 2016). These reporter constructs are based on *cis*-regulatory modules from the *dei* locus (Nachman *et al.* 2015) that were used for driving cytoplasmic GFP expression in the cap and ligament cells of ChOs (*dei^ChO^-GFP*), and cytoplasmic RFP in the attachment cells of ChOs (*dei^attachment^-RFP*) (Figure 1C). For the screening procedure the *dei^ChO^-GFP*, *dei^attachment^-RFP* chromosome was recombined to *ato-Gal4*, which drives expression specifically in the LCh5 lineage, and to *en-gal4*, which drives earlier and prolonged expression in the entire posterior compartment of the segment, including the LCh5 organs. Both of these drivers induce expression in all of the lineage-related cells of the ChO but do not induce expression in the LA cell, which is not derived from the lineage (shown schematically in Figure 5B). Flies from each of these strains were crossed to flies bearing *UAS-RNAi* transgenes from the VDRC collection and the ChO phenotype of the progeny was inspected in whole-mount 2^nd^ instar larvae. At least 10 larvae of each genotype were examined.

A collection of 918 RNAi strains directed against 547 candidate genes (Table S1) was selected and screened with both of the Gal4 drivers. The largest group of genes (240 genes, 379 RNAi lines) among this collection was selected based on gene expression pattern. It consisted of genes reported by (Cachero *et al.* 2011) or (Senthilan *et al.* 2012) to be enriched in ChOs during early stages of embryonic development or in antennal ChOs, respectively. The rest of the genes were selected based on potential functions rather than expression patterns. Since the accessory cells of ChOs are extremely microtubule-rich, we selected 112 genes (188 RNAi lines) identified in FlyMine (http://www.flymine.org) in a search for ‘tubulin-related’ genes. Since ChO morphogenesis in both the embryo and the larva requires extensive cell migration (Inbal *et al.* 2003; Halachmi *et al.* 2016), we selected additional 165 genes (280 RNAi lines) identified in FlyMine using the search term ‘cell migration’. Additional 30 genes (71 RNAi lines) that were identified in previous screens for PNS development (Salzberg *et al.* 1994; Kania *et al.*, 1995; Salzberg *et al.*, 1997), or were identified as being expressed in ChOs in late developmental stages (A. Salzberg, unpublished observations), were also included. When possible, two independent RNAi strains from different libraries (GD and KK) were tested for each gene. RNAi strains identified in the primary large-scale screen were further analyzed using immunohistochemistry on dissected third instar larvae. Complementary RNAi strains from the TRiP collection (Perkins *et al.* 2015) were used for validating the specificity of the RNAi-induced phenotypes.

### Phenotypic grouping

the fluorescent markers used in the screen allowed us to identify phenotypes that could be grouped into three general and not mutually exclusive categories: 1. Loss or gain of GFP or RFP expression, often combined with abnormal morphology of cells. 2. Defective attachment or cell morphology without a major loss of marker expression. 3. Abnormal pattern of cell elongation. We assigned each of the identified genes into one of these three groups based on the most prominent phenotypic feature it presented (Tables 1, 2, and 3).

**Table 1:** Loss or gain of GFP/RFP expression

Gene	CG number	Phenotype	RNAi strain*	Library	*ato* -Gal4	*en*-Gal4	Predicted off targets
*vein*	*CG10491*	Loss of LA cells	109437	KK	—	+	1
*N*	*CG3936*	Loss of CA cells, collapsed cap cells, expansion of the *dei^ChO^*-GFP signal into the region of the sensory unit	100002	KK	+	+	0
			1112	GD	—	—	
*caps*	*CG11282*	Loss of CA cells, collapsed cap cells, increased number of cap cells	3046	GD	+	+	0
			27097	GD	—	—	
			JF02854	TRiP	NT	—	
			JF03418	TRiP	NT	—	
*meru*	*CG32150*	Loss of CA cells	21668	GD	—	—	
			21669	GD	—	+	0
*Dad*	*CG5201*	Loss of CA cells, abnormal organ shape, expansion of the *dei^ChO^*-GFP signal into the region of the sensory unit	42840	GD	—	+	1
			JF02133	TRiP	—	—	
			HMS01102	TRiP	—	—	
*sv*	*CG11049*	Loss of cap and CA cells, or loss of the *dei^ChO^*-GFP *dei^attachment^*-RFP signal	107343	KK	+	+	0
			JF02582	TRiP	—	—	
*pros*	*CG17228*	Expansion of the *dei^ChO^*-GFP expression into the region of the sensory unit	101477	KK	+	+	
			HMJ02107	TRiP	N	+	
			JF02308	TRiP	N	+	

* VDRC or BDSC transformant ID, NT – not tested.

Table 1 lists the seven genes identified in the screen whose knockdown by RNAi led to loss or expansion of the *dei^ChO^-GFP* and/or *dei^attachment^-RFP* reporters. The RNAi strains directed against each of the genes, the phenotype they caused, and the ability of each RNAi strain to cause a phenotype when expressed under the regulation of *ato-Gal4* and *en-Gal4* are listed. The number of predicted off targets is indicated for RNAi strains whose phenotypes were not reproduced by additional RNAi strains directed against the same gene.

**Table 2:** Defective attachment or cell morphology

Gene	CG number	Phenotype	RNAi strain*	Library	*ato -Gal4*	*en-Gal4*	Predicted off targets
*Egfr*	*CG10079*	Small CA cells that express low levels of the *dei^attachment^*-RFP marker, thinning of the cap cells close to the cap/CA attachment site	107130	KK	—	+	
			43267	GD	NT	—	
			43268	GD	—	—	
			JF01696	TRiP	NT	+	
			JF01083	TRiP	NT	—	
			JF01084	TRiP	NT	—	
			JF01368	TRiP	NT	+	
*cpo*	*CG43738*	Small, slightly elongated CA cells that express very low levels of the *dei^attachment^*-RFP marker, thinning of the cap cells close to the cap/CA attachment site	14385	GD	—	+	610**
			JF02996	TRiP	—	—	
*CG13653*	*CG13653*	Small CA cells that express low levels of the *dei^attachment^*-RFP marker, thinning of the cap cells close to the cap/CA attachment site	15436	GD	—	+	0
			106259	KK	—	—	
*fry*	*CG32045*	Small, slightly elongated CA cells	40309	GD	—	+	633**
			103569	KK	—	—	
*ed*	*CG12676*	Small CA cell, occasional detachment of cap cells (mostly mild phenotypes)	104279	KK	NT	+	1
			3087	GD	NT	Very mild phenotype	0
			938	GD	NT	—	
*Eb1*	*CG3265*	Small, slightly elongated CA cells that express very low levels of the *dei^attachment^*-RFP marker	24451	GD	—	+	0
			HM05093	TRiP	—	—	
*WASp*	*CG1520*	Small, slightly elongated CA cells that express very levels of the *dei^attachment^*-RFP marker	13757	GD	NT	+	0
			108220	KK	NT	mild phenotype	0
*pyr*	*CG13194*	Slightly elongated CA cells, shorter than normal cap cells, longer than normal ligament cells	36524	GD	—	+	
			36523	GD	—	+	
*sr*	*CG7847*	Defective CA cells, detachment of cap cells, longer than normal ligament cells	105282	KK	+	Lethal	
			9921	GD	—	+/−	
			JF02781	TRiP	—	+ /-	
*mys*	*CG1560*	Abnormal connection between the cap and CA cells (detachment of the cap cells or thinning of the cap cells in the cap/CA attachment region). Abnormally short cap cells and longer than normal ligament cells	29619	GD	+	Lethal	
			29620	GD	—	—	
			103704	KK	—	Very mild phenotype	
			HMS00043	TRiP	+	Lethal	
			JF02819	TRiP	NT	—	
*sens*	*CG32120*	Uneven length of cap cells. Expansion of the *dei^ChO^*-GFP expression into the region of the sensory unit	106028	KK	—	+	0
*Rac 1*	*CG2248*	Uneven length of cap cells. Long ligament cells	49246	GD	NT	—	
			49247	GD	—	+	1
			50349	GD	—	+/−	
			50350	GD	+/−	—	
*raw*	*CG12437*	Uneven length of cap cells. Expansion of the *dei^ChO^*-GFP expression into the region of the sensory unit	24532	GD	—	+	0
			101255	KK	—	—	
			JF01382	TRiP	NT	—	

* VDRC or BDSC transformant ID.

** High number of off targets.

NT – not tested.

Table 2 lists the thirteen genes identified in the screen whose knockdown by RNAi led to defective pattern of attachment or cell morphology. The RNAi strains directed against each of the genes, the phenotype they caused, and the ability of each RNAi strain to cause a phenotype when expressed under the regulation of *ato-Gal4* and *en-Gal4* are listed. The number of predicted off targets is indicated for RNAi strains whose phenotypes were not reproduced by additional RNAi strains directed against the same gene.

**Table 3:** Abnormal pattern of cell elongation

Gene	CG number	Phenotype	RNAi strain*	Library	*ato -Gal4*	*en-Gal4*	Predicted off targets
*αTub85E*	*CG9476*	Short cap cells, longer than normal ligament cells, long CA cells	103202	KK	+	Lethal	0
			HM04009	TRiP	NT	—	
*αTub67C*	*CG8308*	Short cap cells, long ligament cells.	108044	KK	—	+	1
*αTub84B*	*CG1913*	Short cap cells, long CA cells	52345	GD	—	+	
			JF01373	TRiP	NT	+	
*βTub60D (β3Tub)*	*CG3401*	Short cap cells, long CA cells	34607	GD	+	Lethal	
			102052	KK	—	—	
*βTub56D (β1Tub)*	*CG9277*	Short cap cells, longer than normal ligament cells	24138	GD	+	+	
			109736	KK	+	+	
*βTub97EF*	*CG4869*	Short cap cells, long CA, cells	105075	KK	+	+	1
*βTub85D*	*CG9359*	Short cap cells, long CA, cells	24144	GD	NT	+	4
			109590	KK	—	—	
*Tbce*	*CG7861*	Short cap cells, long CA cells	105246	KK	—	+	1
*CCT8*	*CG8258*	Short cap cells, long CA cells	103905	KK	+	—	1
			45790	GD	—	—	
*shot*	*CG18076*	Short cap cells, long CA cells, long ligament cells, detachment between cap and CA cells	JF02971	TRiP	+	Lethal	
			GL01286	TRiP			
*tx / dei*	*CG5441*	Short cap cells, longer than normal ligament cells	37629	GD	—	+	
			37630	GD	—	+	
			102831	KK	—	—	
			JF01995	TRiP	NT	+/−	

* VDRC or BDSC transformant ID, NT – not tested.

Table 3 lists the eleven genes identified in the screen whose knockdown by RNAi led to abnormal pattern of ChO cell elongation. The RNAi strains directed against each of the genes, the phenotype they caused, and the ability of each RNAi strain to cause a phenotype when expressed under the regulation of *ato-Gal4* and *en-Gal4* are listed. The number of predicted off targets is indicated for RNAi strains whose phenotypes were not reproduced by additional RNAi strains directed against the same gene.

### Loss or gain of GFP or RFP expression

**Figure 2:**
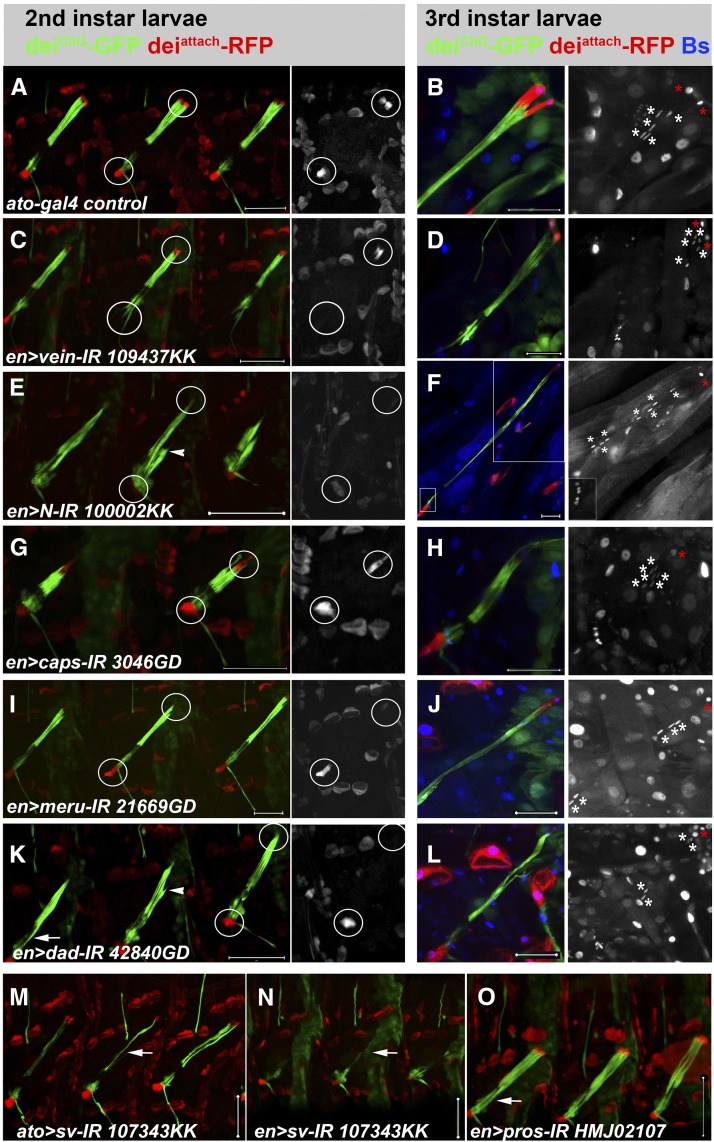
Loss or gain of GFP/RFP expression. (A-L) LCh5 organs of control and knockdown 2^nd^ and 3^rd^ instar larvae visualized by the expression of the *dei^ChO^-GFP* (green) and *dei^attachment^-RFP* (red) reporters. The ChOs of third instar larvae were additionally immunostained with anti-Bs antibody (blue, shown separately in the insets). The CA cells and the LA cell are circled (A, C, E, G, I, K) and shown separately in the insets. In (B, D, F, H, J, L) the red asterisks mark the CA cells’ nuclei and the white asterisks mark the cap cells’ nuclei. (A-B) *ato-gal*, *dei^ChO^-GFP*, *dei^attachment^-RFP* larvae. (C-D) larvae expressing an inverted repeat construct (IR) directed against *vein* under the regulation of *en-gal*. The LA cell fails to form. (E-F) larvae expressing an IR construct directed against *Notch* under the regulation of *en-gal*. Note the loss of CA cells and the collapse of cap cells (arrowhead). Seven cap cells and a single CA cell are evident in the shown 3^rd^ instar larva (F). The inset on the right shows a close-up view of the boxed area in F. (G-H) larvae expressing an IR directed against *caps* under the regulation of *en-gal*. (G) One CA cell is lost and the LCh5 organ appears collapsed. Six cap cells and a single CA cell are evident in the shown 3^rd^ instar larva (H). (I-J) larvae expressing an IR directed against *meru* under the regulation of *en-gal*. Note the loss of one CA cell and the abnormal position of some of the cap cells’ nuclei. (K-L) larvae expressing an IR directed against *Dad* under the regulation of *en-gal4*. The loss of one or two CA cells and concomitant collapse of cap cells (arrowhead) is evident. (M-O) LCh5 organs of knockdown 2^nd^ larvae visualized by the expression of the *dei^ChO^-GFP* (green) and *dei^attachment^-RFP* (red) reporters. (M-N) Larvae expressing an IR directed against *sv* under the regulation of *ato-gal4* (M) or *en-gal4* (N). Note the loss of cap-specific GFP expression (arrows). (O) A larva expressing an RNAi construct directed against *pros*. Note the expansion of the GFP signal into the region of the sensory unit (arrow). Scale bars = 50 μm.

As outlined in Table 1 and Figure 2, seven genes were identified whose knockdown by RNAi led to a loss of GFP or RFP expression from specific ChO cells. The loss of marker expression could reflect a genuine loss of specific cell types, cell fate transformation, or specific loss of *dei* expression. Similarly, expansion of GFP/RFP expression could reflect gain of cells, cell fate transformation, or ectopic expression of the *dei* gene. Although the loss of marker expression does not necessarily reflect a true loss of specific cell types, we refer to the phenotypes as ‘loss of cells’ for the sake of simplicity, and group the phenotypes according to the type of the affected cell/s.

#### Loss of LA cells:

The phenotype caused by knocking down *vein* (*vn*) expression under the regulation of *en-Gal4* was unique. *vn* is the only gene identified whose knockdown within the ChO lineage led to a non-autonomous loss of the LA cell (Figure 2C-D). The LA cell is recruited from the epidermis via an EGFR-mediated pathway; the current observation validates the previously suggested notion that Vn is the ligand secreted by the ligament cells (Inbal *et al.* 2004). As a consequence of reducing Vn secretion from the ligament cells by means of *vn* RNAi expression, the EGFR is not activated in the target epidermal cell and therefore, LA cell differentiation does not occur. The observed *vn* RNAi phenotype also suggests that a crosstalk between the ligament cells and the LA cell is required for the convergence of the ligament cells’ migrating tips onto a narrow attachment site. In the absence of a LA cell, the ligament cells’ tips extend in different directions (Figure 2C).

#### Loss of CA cells:

Expressing RNAi constructs directed against three genes, *capricious* (*caps*), *Notch* (*N*) and *meru*, led to a loss of at least one of the two CA cells (Figure 2E-J), which was often accompanied with a collapse of the cap cells. In order to better characterize the phenotypes and distinguish between CA cell loss and cell fate transformation, we counted the number of cap and CA cells present in the affected LCh5 organs using anti-Blistered (Bs) immunostaining. This analysis demonstrated that in the *N* and *caps* knockdown larvae, the loss of CA cells was consistently accompanied by an increase in the number of cap cells. Whereas the LCh5 organs of control larvae contained five cap cells and two CA cells each, the LCh5 of *N*- or *caps*-RNAi larvae consisted of one CA and six (or, occasionally, seven) cap cells (Figure 2E-H). These results suggest that the activity of both *N* and *caps* is required for the correct specification of CA *vs.* cap cell-fate by influencing the asymmetric division of the secondary ChO precursor that gives rise to the cap and CA cells. This finding corroborates findings of a previous RNAi screen that identified *caps* as a gene affecting asymmetric cell division in the external sensory lineage (Mummery-Widmer *et al.* 2009).

Unlike *N* and *caps*, the knockdown of *meru* led to the loss of one CA cell with no concomitant increase in the number of cap cells (Figure 2I-J). The *meru* gene was identified by Reeves and Posakony (Reeves and Posakony 2005) as a direct target of the proneural genes and was implicated in the sensory perception of pain by (Neely *et al.* 2010). More recently, (Banerjee *et al.* 2017) have identified Meru as a modulator of cell polarity that connects planar cell polarity with apical-basal polarity during asymmetric cell divisions within the external sensory organ lineage. The identification of *meru* in the current screen points to a possible role of *meru* in the ChO lineage as well. Whether its role in the internal sensory (ChO) lineage is similar to its role in the external sensory lineages remains to be elucidated. A more severe and variable phenotype was caused by down-regulating the *daughters against DPP (Dad)* gene within the posterior compartment of the segment. *Dad* downregulation led to loss of the two CA cells, often collapse of the cap cells and expansion of the *dei*^ChO^-GFP expression into the region of the sensory unit (Figure 2K-L).

#### Loss of cap and CA cells:

The expression of *shaven* (*sv*)-RNAi under the regulation of either *ato*-gal4 or *en*-gal4 caused a severe loss of cap and CA cells that was not accompanied by an obvious increase in the number of other types of cells (Figure 2M-N). This observation suggests that Sv is required for the differentiation and/or survival of the cap and cap-attachment cells. Interestingly, the *sv* gene is required for the differentiation of shaft cells, which are equivalent to the cap cells in the adult external sensory (ES) lineages (Fu *et al.* 1998; Kavaler *et al.* 1999).

#### Expansion of GFP expression:

The expression of *prospero* (*pros*)-RNAi under the regulation of either *ato-gal4* or *en-gal4* led to an expansion of the *dei*^ChO^-GFP expression into the region of the sensory unit (Figure 2O). This phenotype could indicate that loss of *pros* expression causes the scolopale cell, the only *pros*-expressing cells in the ChO lineage, to acquire an accessory (ligament or cap) cell identity. As mentioned above, the knockdown of *Dad* often led to a similar expansion of GFP expression into the sensory unit (Figure 2K), and so did the knockdown of *senseless* (see below, 3N).

### Defective attachment or cell morphology

In wildtype larvae, the cap cells are stretched between the scolopale cells and the CA cells. During larval growth, the CA cells grow dramatically, extending numerous tubulin-rich extensions and forming a wide integrin-rich junction with the attached cap cells (Halachmi *et al.* 2016; Greenblatt Ben-El *et al.* 2017). These morphological changes are likely required for adjusting the ability of the CA cells to anchor the cap cells and remain attached to the cuticle under conditions of increasing mechanical stresses. Ten genes were identified in the screen whose knockdown caused an abnormal pattern of cap/CA cell attachment. Three additional genes affected the cap cells on their scolopale-facing side (Table 2).

#### Defective attachment between the cap and CA cell:

**Figure 3:**
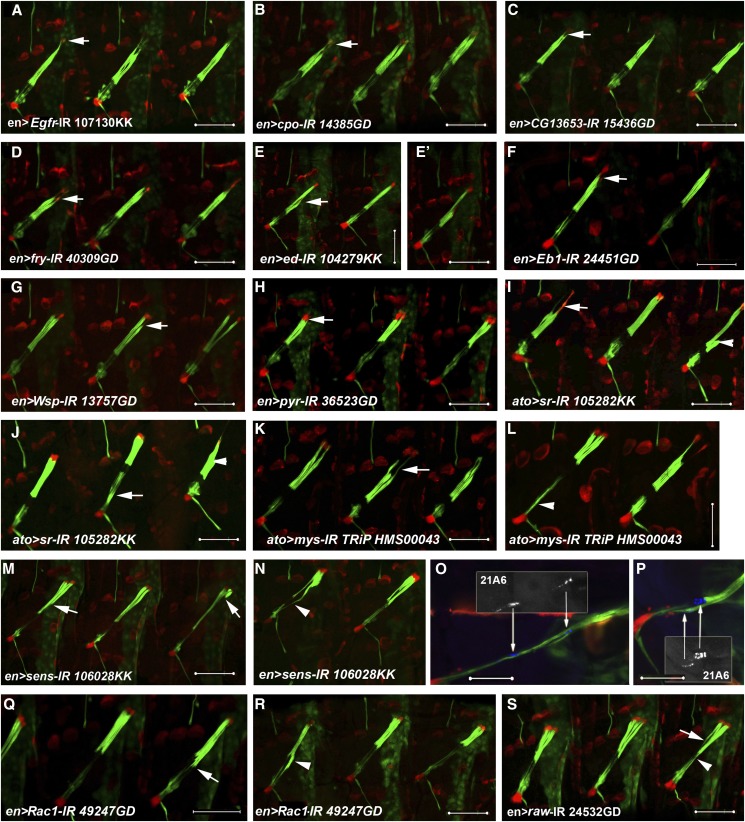
Defective attachment or cell morphology. (A-S) LCh5 organs of knockdown 2^nd^ (A-N, Q-S) and 3^rd^ (O-P) instar larvae visualized by the expression of the *dei^ChO^-GFP* (green) and *dei^attachment^-RFP* (red) reporters. The ChOs of third instar larvae were additionally immunostained with Mab 21A6 (blue) which marks the scolopale cells. (A-C) Larvae expressing an RNAi construct directed against *Egfr* (A), *cpo* (B) or *CG1365* (C) under the regulation of *en-gal*. The arrows point to the abnormally small CA cells and the pointed appearance of the cap cells near the attachment site. (D) A larva expressing an RNAi construct directed against *fry*. The arrow points to the slightly elongated CA cells. (E, E’) Larvae expressing an RNAi construct directed against *ed*. The arrow points to detached and collapsed cap cells. (F-H) Larvae expressing RNAi constructs directed against *Eb1* (F), *Wsp* (G) or *pyr* (H) under the regulation of *en-gal*. The arrows point to the abnormally shaped CA cells. (I-J) LCh5 organs of larvae expressing *sr*-specific RNAi under the regulation of *ato-gal4*. The arrowheads in (I-J) point to detached cap cells; the arrows point to abnormally elongated CA cell (I) or ligament cell (J). (K-L) LCh5 organs of larvae expressing *mys*-specific RNAi under the regulation of *ato-gal4*. The arrow in (K) points to the abnormal thinning of the cap cells close to the attachment site. The arrowhead in (L) points to abnormally elongated ligament cells. (M-P). 2^nd^ (M-N) and 3^rd^ (O-P) Larvae expressing *sens*-specific RNAi under the regulation of *en-gal4*. Note the loss of alignment of the cap cells on their ventral side (arrows in M) and the abnormal expansion of the GFP marker into the region of the scolopale cell (arrow in N). The 21A6 staining reveals the abnormal position of the scolopale cells (arrows in O-P). (Q-S) 2^nd^ instar larvae expression RNAi construct directed against *Rac1* (Q-R) or *raw* (S). The arrows point to the loss of alignment of the cap cells on their ventral side; the arrowheads point to the abnormal extension of the GFP signal into the region harboring the sensory unit. Scale bars = 50 μm.

Down-regulation of the *Drosophila* EGF-receptor gene, *Egfr*, within the *en* domain resulted in the development of small, often slightly elongated, CA cells that expressed lower levels of the *dei^attachment^*-RFP marker as compared to control larvae. The contact area between the affected CA cells and the attached cap cells was greatly reduced and the bundle of five cap cells appeared abnormally thin near the attachment site (Figure 3A). The LA cell, which depends on Egfr activity for its development, does not originate from the *en* domain and thus could develop properly in the *en-Gal4/Egfr-IR* larvae.

The expression of RNAi constructs directed against six additional genes caused a *Egfr*-like phenotype: *couch potato* (*cpo*), which encodes for an RNA binding protein, *CG13653*, a gene with unknown function, *furry* (*fry*), which encodes for an actin cytoskeleton regulator, *echinoid* (*ed*), which encodes for a homophilic cell adhesion molecule, *Eb1*, which encodes for a microtubule-associated protein, and *WASp* (*Wsp*) the fly homolog of the Wiskott-Aldrich Syndrome family of actin nucleation factors (Figure 3B-G). The expression of RNAi construct directed against *pyramus* (*pyr*), which encodes for one of the three known *Drosophila* Fibroblast Growth Factor (FGF) ligands led to the development of abnormally shaped CA cells and slightly elongated ligament cells (Figure 3H).

Two other genes found to be important for proper attachment between the cap and CA cells were *stripe* (*sr*), which encodes for an early growth response-like transcription factor, and *myospheroid* (*mys*), which encodes for the prevalent variant of beta-integrin (βPS). Sr has been previously shown to be required for CA cell differentiation in the embryo (Inbal *et al.* 2004). Here we show that during larval stages the Sr-deficient CA cells fail to anchor the cap cells properly, leading to their detachment and collapse (Figure 3I-J). The phenotype of the *ato-Gal4/sr-IR* larvae also validates the notion that the LA cell depends on the *autonomous* activity of Sr (Inbal *et al.* 2004) and could, therefore, develop properly in the *ato-Gal4/sr-IR* larvae. In addition to the defects in cap cell attachment, the *sr* knockdown larvae occasionally presented elongated CA or ligament cells (Figure 3J).

Reducing the level of *mys* expression had no major effect on the differentiation of the CA cells, as suggested by their normal size and overall morphology as well as the normal level of *dei^attachment^-RFP* expression they presented. However, the loss of βPS integrin led to detachment and collapse of the cap cells. In segments in which the cap cells remained attached, the contact area between the cap and CA cell was greatly reduced and the cap cell appeared much thinner than normal close to the cap/CA contact point (Figure 3K-L). Occasionally, the *mys* knockdown larvae presented elongated ligament cells in addition to the defects in cap cell attachment (Figure 3L). This observation supports the idea that cap cell elongation depends on integrin-based interaction with the extracellular matrix (Greenblatt Ben-El *et al.* 2017).

#### Abnormal alignment or attachment of the cap cells on their scolopale-facing side:

The knockdown of three genes, *senseless* (*sens*), *raw* and *Rac1* affected the cap cells on their ventral side where they normally attach to the scolopale cells. *sens*, which encodes for a zinc finger transcription factor, is an important regulator of neurogenesis in the embryonic PNS, where it is required for enhancement and maintenance of proneural gene expression in the sensory organ precursors (Salzberg *et al.* 1994; Nolo *et al.* 2000). Unlike normal larval ChOs, in which the ventral tips of all five cap cells are aligned, the cap cells of *sens*-depleted larvae vary in length and often appear shorter and detached on their ventral side (Figure 3M-N). In other segments, the cap cell-specific GFP signal expanded into the scolopale cell (Figure 3N). A closer examination of the affected organs in 3^rd^ instar larvae demonstrated that shorter cap cells remained attached to scolopale cells that were located in abnormal dorsal positions, possibly reflecting defects in ChO cell migration. The expansion of the GFP signal into the scolopale cell suggests a partial scolopale-to-cap cell fate transformation (Figure 3O-P).

In *raw* deficient larvae, the cap cells are of varying lengths and some of them seem to extend into the region normally occupied by the scolopale cells (Figure 3S). Raw, a membranous protein, was previously shown to be involved in cell movement, elongation and ensheathment *e.g.*, (Jack and Myette 1997; Blake *et al.* 1998, 1999; Byars *et al.* 1999; Bates *et al.* 2008; Jemc *et al.* 2012), thus the observed phenotype could reflect defects in the interactions between the cap and scolopale cells that lead to abnormal contact between the two cell types. In a previous PNS screen, insertional mutations in the *raw/cyr* gene caused a ChO phenotype of darkly stained (MAb22C10) elongated neuronal cell bodies, thicker than normal axon bundles and mild pathfinding defects (Kania *et al.* 1995; Prokopenko *et al.* 2000). Irregularities in cap cell alignment and occasional expansion of the GFP signal into the scolopale cell was also evident in *Rac1* knockdown larvae (Figure 3Q-R). Rac1, a small GTPase is involved in regulating the dynamic rearrangements of the actin cytoskeleton and was shown to play a role in peripheral glia migration, nerve ensheathment and axon outgrowth (Luo *et al.* 1994; Sepp 2003). Additional phenotypes observed in the *Rac1* knockdown larvae were longer than normal ligament cells and detachment of the cap from the CA cells (Figure 3R). The identification of *Rac1* as well as *fry*, *WASp* and *shot* (see below) point to the importance of the actin cytoskeleton in ChO morphogenesis.

### Abnormal pattern of cell elongation

**Figure 4:**
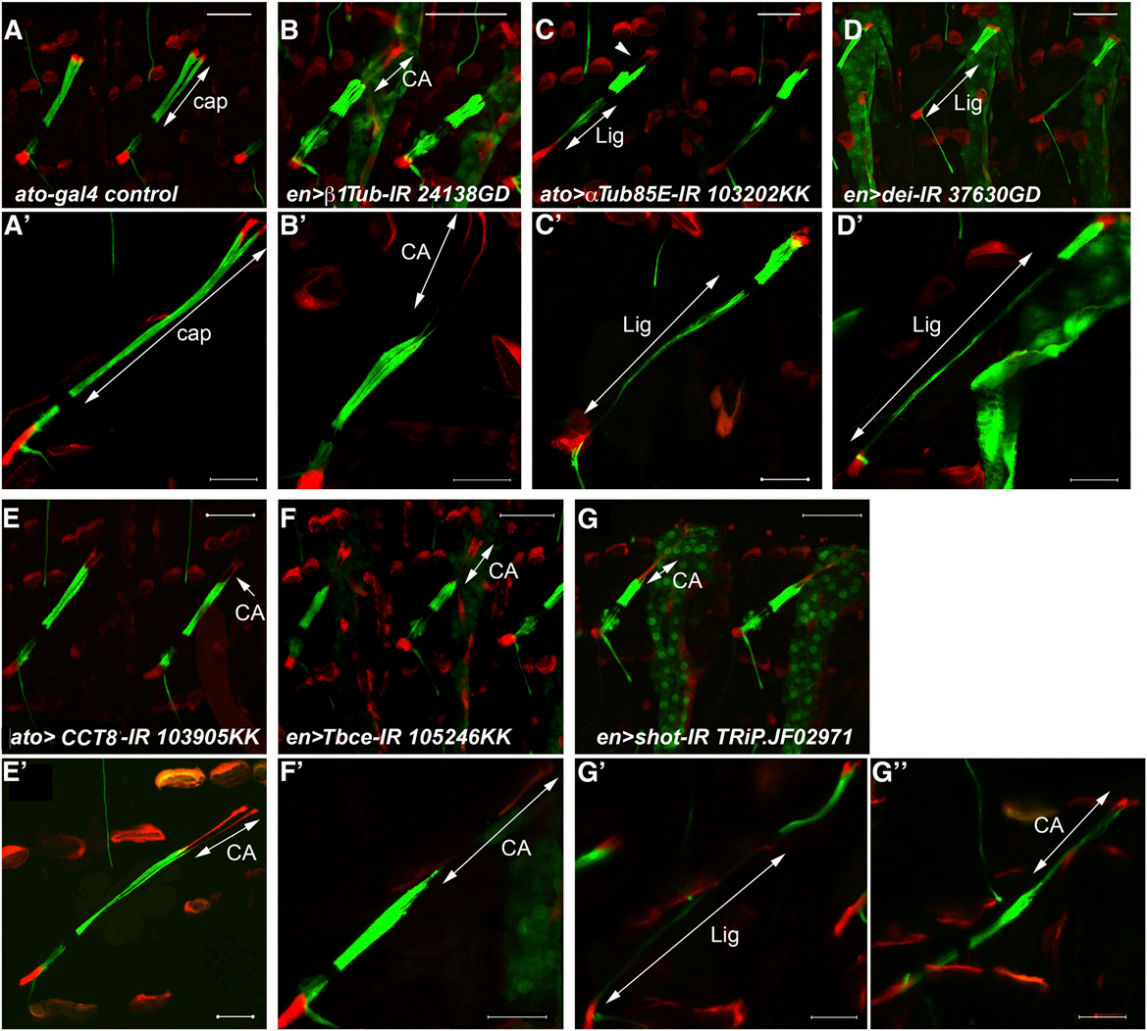
Abnormal pattern of cell elongation. LCh5 organs of 2^nd^ (A-G) and 3^rd^ (A’-G’’) larvae visualized by the *dei^ChO^-GFP* (green) *dei^attachment^-RFP* (red) reporters. (A, A’) Control *ato-gal4* larvae; the normal length of the cap cells is indicated by arrows. (B, B’) Larvae expressing β*1Tub* RNAi under the regulation of *en-gal4*; the abnormally long CA cells are indicated by arrows. (C, C’) Larvae expressing *αTub85E* RNAi under the regulation of *ato-gal4*; the abnormally long ligament cells are indicated by arrows. The arrowhead in C points to an elongated CA cell. (D, D’) Larvae expressing *dei* RNAi under the regulation of *en-gal4*; the abnormally long ligament cells are indicated by arrows. (E, E’) *ato-gal4/CCT8* RNAi larvae. The arrows point to the elongated CA cells. (F, F’) *Tbce* RNAi transgene driven by *en-gal4*; the abnormally long CA cells are indicated by arrows. (G, G’, G’’) Larvae expressing *shot* RNAi transgene under the regulation of *en-gal4*. 2^nd^ instar larvae present a long CA cell phenotype (G). 3^rd^ instar larvae present variable abnormal elongation of ligament (G’) and CA (G’’) cells. The abnormally elongated cells are indicated by arrows. Scale bars = 50 μm.

During larval growth, the LCh5 organ, which is anchored on both its sides to the cuticle, stretches and elongates from approximately 70 microns at the end of embryogenesis to more than 300 microns at the 3^rd^ instar larva. Normally, most of this elongation is attributed to the cap cell, which increases its length nearly 13-fold and comprises 65–70% of the entire organ length. We have identified 11 genes whose knockdown led to an abnormal pattern of cell elongation within the ChO (Table 3). In larvae expressing RNAi constructs against any of the identified genes, the cap cells were shorter than normal, whereas the ligament cells and/or the CA cells, were longer than normal (Figure 4). The total length of the organ did not change. Ten of the genes included in this phenotypic category encode for different variants of α and β tubulin and for other types of microtubule-associated proteins: seven tubulin genes, two chaperones (*tubulin-specific chaperone E* (*Tbce*) and *CCT8*), and the spectraplakin-encoding gene *shortstop* (*shot*). The 11^th^ gene in this phenotypic group encodes for the basic helix-loop-helix transcription factor Taxi wings/Delilah (Dei). The knockdown of three additional genes included in other phenotypic categories, *sr*, *mys* and *Rac1*, lead to abnormal elongation of the ligament cells (see Figure 3J, L, Q-R, respectively).

α and β tubulin are encoded in the *Drosophila* genome by a small gene family comprised of five genes for α tubulins and five genes for β tubulins (Sánchez *et al.* 1980; Gramates *et al.* 2017). Although RNAi transgenes directed against seven of these genes (*αTub85E*, *αTub67C*, *αTub84B*, *βTub60D*, *βTub56D*, *βTub97EF*, *βTub85D*) caused defects in LCh5 cell elongation, we suspect that some of the phenotypes were caused by off-targeting effects and do not reflect a genuine requirement for that specific tubulin isoform. Based on information provided by the VDRC, off-targeting is common among RNAi transgenes directed against the various α tubulin isoforms and among RNAi transgenes directed against the various β tubulin isoforms (Table S2). We therefore refer here only to the two major tubulin isoforms that are expressed within the ChO, namely *αTub85E* and *βTub56D* (*β1 tub*).

**Figure 5:**
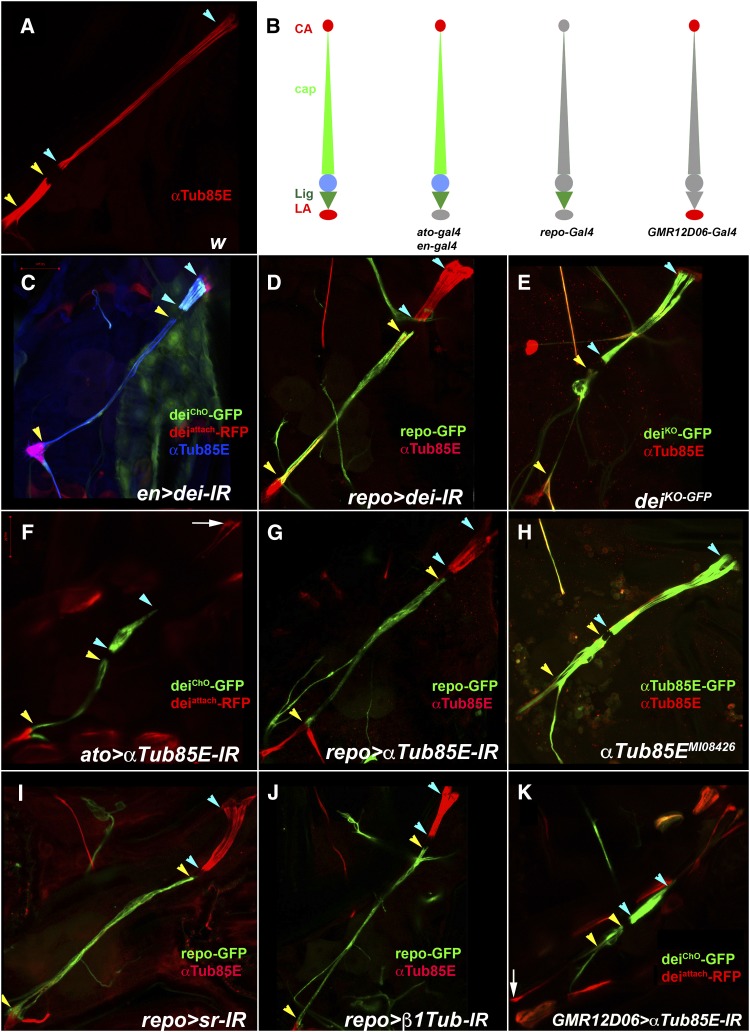
Genes required for keeping the ligament cells short. (A) An LCh5 organ of a wildtype larva visualized by anti-αTub85E staining. (B) A schematic illustration of an LCh5 organ and the expression pattern of the various drivers used in this study. The CA and LA cells are depicted in red, the cap cell in light green, the ligament cell in dark green, and the sensory unit is represented by a blue circle. For each Gal4 driver colored symbols represent the expressing cells, whereas the gray symbols denote cells which do not express the driver. (C-K) Each micrograph shows a single LCh5 organ of a third instar larva. The light blue arrowheads delineate the length of the cap cells; the yellow arrowheads delineate the length of the ligament cells in each organ. (C-D) Larvae in which the expression of *dei* was knocked down under the regulation of *en-Gal4* (C) or specifically in the ligament cells under the regulation of *UAS-CD8-GFP*; *repo-Gal4* (D). (E) A homozygous *dei^KO-GFP^* larva. (F-G) Larvae in which the expression of *αTub85E* was knocked down under the regulation of *ato-Gal4* (F) or specifically in the ligament cells under the regulation of *UAS-CD8-GFP*; *repo-Gal4*. The white arrow in F points to the dorsal tip of the elongated CA cells. (H) A homozygous *αTub85E^MI08426-GFSTF.0^* larva. (I-J) Larvae in which the expression of *sr* (I) or β1*Tub* (J) was knocked down under the regulation of *UAS-CD8-GFP*; *repo-Gal4*. (K) A Larva in which the expression of α*Tub85E* was knocked down specifically in the attachment cells under the regulation of *GMR12D06-Gal4*. The white arrow points to the ventral tip of the elongated LA cell.

Interestingly, the knockdown of α and β tubulin genes led to distinguishable phenotypes. Despite the fact that both *αTub85E* and *β1Tub* are expressed in all of the accessory and attachment cells, down-regulation of α*Tub85E* led to shortening of the cap cells and concomitant elongation of, primarily, the ligament cells, whereas down-regulation of *β1Tub* led to shortening of the cap cells and elongation of the CA cells (Figure 4B-C). By the time the affected larvae reached the 3^rd^ instar larval stage, the CA cells of the *αTub85E* knockdown larvae were often elongated as well (see Figure 5F), yet the phenotypic difference between the *α* and *β* gene was still evident. In contrast to the *αTub85E* knockdown larvae, in the *β1-tub* knockdown 3^rd^ instar larvae the ligament cells were not elongated (Figure 4B’). Knocking down the expression of *CCT8*, *Tbce*, or *shot* led to a *βT*ub-like phenotype, whereas knocking down the expression of *dei* led to a pronounced *αTub85E*–like phenotype (Figure 4D-G). The ligament cells of the *shot* knockdown larvae were occasionally elongated as well (Figure 4G’).

### Keeping the ligament cells short

The pronounced cell-elongation phenotypes observed upon knocking down the expression of either *dei* or *αTub85E* was somewhat surprising since, previously, we have shown that a deletion of the *αTub85E* locus did not lead to any obvious defects in ChO’s morphology in late embryos (Klein *et al.* 2010). Similarly, examination of the LCh5 organs of *dei* deficient embryos did not reveal any abnormal phenotypes, suggesting that Dei does not play a critical role in embryonic ChO development (A.Salzberg unpublished data). The current observations, however, implicate both *αTub85E* and *dei* in ChO morphogenesis and suggest for the first time that their loss affect the ability of the ChO cells to elongate properly in response to developmental organ stretching.

*dei* and *αTub85E* share the same expression pattern within the ChO, both being expressed in the cap, ligament, CA and LA cells. Thus, the excessive elongation of the ligament cells caused by their loss of function could stem from the inability of the cap cells to elongate properly in response to organ’s stretching, or from the inability of the ligament cells to resist stretching and remain short. In order to test whether *dei* and *αTub85E* are required for preventing ligament cell elongation, we down-regulated their expression specifically in the ligament cells under the regulation of *repo–gal4*. The ligament-specific knockdown of either *αTub85E* or *dei* resulted in extremely elongated ligament cells, shorter than normal cap cells, and normally shaped CA and LA cells (Figure 5D, G). These observations indicate that both *αTub85E* and *dei* are critical for the development of ligament cells that are able to remain short during organ elongation. A cap cell-specific Gal4 driver (currently not available) is needed for establishing whether these genes are additionally required for the inherent ability of the cap cells to elongate properly. To validate the RNAi-induced phenotypes of *αTub85E* and *dei*, and to examine the effects of eliminating or reducing their expression from the entire ChO, including the LA cell, we examined the phenotypes of larvae homozygous for the viable weak hypomorphic allele *αTub85E^MI08426-GFSTF.0^*, or larvae homozygous for a *dei* null allele we have generated. Both of the mutants exhibited elongated ligament cells, validating the role of these genes in keeping the ligament cells short (Figure 5E, H). The *αTub85E^MI08426-GFSTF^* larvae displayed in addition slightly elongated LA cells (Figure 5H).

Although *dei* affects ligament cell elongation similarly to *αTub85E*, its effect is probably not mediated through downregulation of *αTub85E* expression as suggested by the persistent *αTub85E* expression in *dei* knockdown or knockout larvae (Figure 5C-E). Another transcription factor identified in the current screen, Sr, was previously found to be a positive regulator of *αTub85E* expression in the ligament cells during embryogenesis (Klein *et al.* 2010). The role of Sr in post-embryonic morphogenesis of the ChO could not be deduced from phenotypic analyses of *sr* mutants due to embryonic lethality, however, as a positive regulator of *αTub85E*, Sr is expected to affect ligament cell elongation. The occasional elongation of ligament cells seen in *ato-gal4*/*sr-RNAi* larvae (Figure 3J) supports such a notion. To further test whether Sr is essential for the development of ligament cells that are resistant to stretching, we knocked down *sr* expression specifically in the ligament cells and examined the ChOs of 3^rd^ instar larvae. As shown in Figure 5I, the knockdown of *sr* within the ligament cells caused a loss of αTub85E expression and extensive elongation of these cells, comparable to the *αTub85E* knockdown phenotype. This observation indicates that Sr activity is critical for the development of ligament cells that avoid cell elongation, possibly through its positive effect on αTub85E expression.

Among the ChO cells, the ligament cells seem the most sensitive to the loss of αTub85E, as they abnormally elongate upon any reduction in its expression levels, while other cells maintain their normal length. In contrast to αTub85E, when *β1Tub* was knocked-down under the regulation of *en-gal4* or *ato-gal4*, the CA cells, rather than the ligament cells, were abnormally long, suggesting the hypothesis that this tubulin may be required for maintaining rigid attachment cells. However, to the best of our knowledge, *β1Tub* is the only *β*-tubulin isotype expressed at high levels in the ligament cells. Thus, if this tubulin indeed affects attachment cell rigidity, it is expected to affect similarly the properties of the ligament cells. Indeed, when we knocked down the expression of *β1Tub* specifically in the ligament cells, it led to their extreme elongation (Figure 5J). Similarly, when we knocked down *αTub85E* specifically in the CA and LA cells, under the regulation of the *GMR12D06-GAL4* driver, it resulted in extreme elongation of these attachment cells (Figure 5K). Altogether these observations suggest that both αTub85E and β1Tub are required for preventing cell elongation of both the ligament cells and the attachment cells, but the attachment cells are more sensitive to the loss of β1Tub whereas the ligament cells are more sensitive to the loss of αTub85E.

## Discussion

The proprioceptive larval ChOs respond to mechanical stimuli generated by muscle contractions and consequent deformations of the cuticle. Thus, their function likely depends on the correct mechanical properties of their accessory (cap and ligament) and attachment (CA and LA) cells that transform the deformation from the cuticle to the sensory neuron. Here we describe a genetic screen that focused, for the first time, on the development of the ChO accessory and attachment cells, rather than the sensory unit itself.

**Figure 6:**
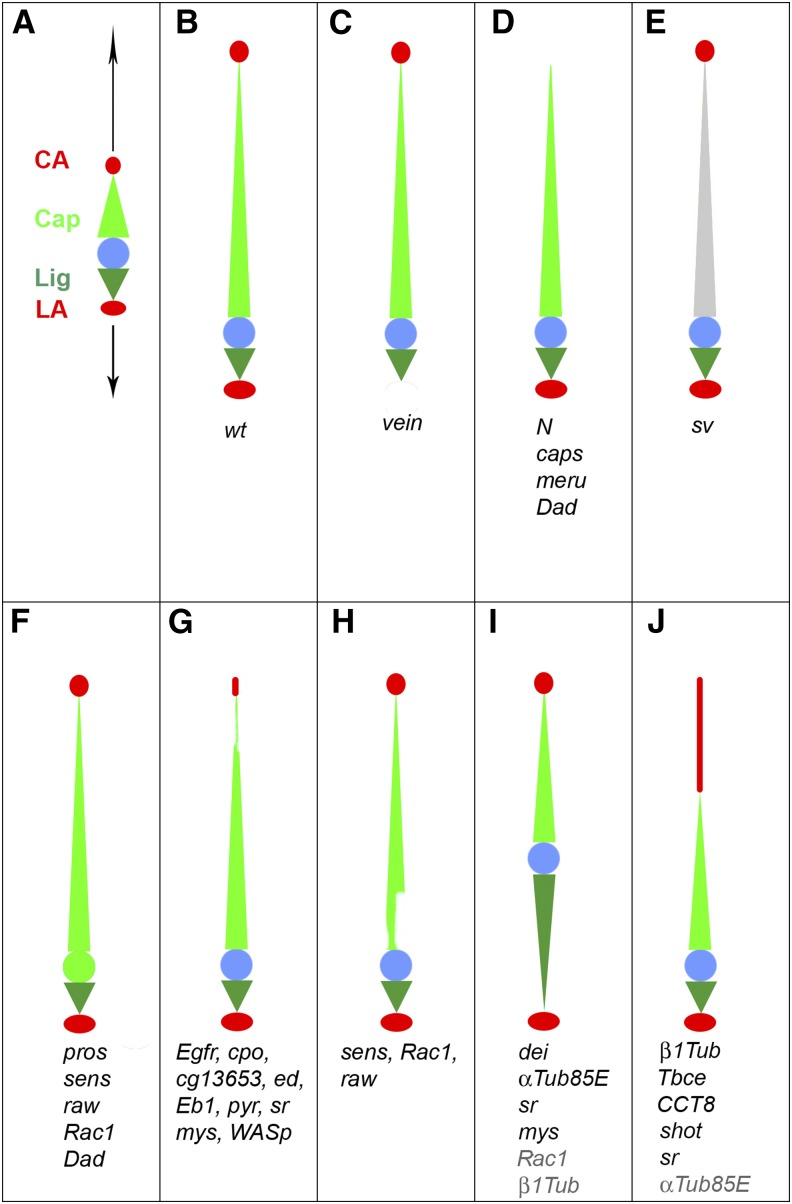
Schematic illustration of the ChO phenotypes and candidate genes identified in the screen. (A-B) Normal ChOs in the embryo (A) and 2^nd^ or 3^rd^ instar larva (B). Here and in all other panels the CA and LA cells are depicted in red, the cap cell in light green, the ligament cell in dark green, and the sensory unit is represented by a blue circle. (A) At the end of embryogenesis, the length of the ChO is approximately 70 μm. Due to larval growth, the attachment cells of the organ are pulled away from each other and the organ stretches. (B) Normally, the only cell type that elongates significantly is the cap cell. (C) Downregulation of *vn* in the ChO lineage prevents the recruitment of LA cell. (D) Downregulation of *N*, *caps*, *meru* or *Dad* leads to loss of CA cells. (E) Knockdown of *sv* interferes with normal cap cell development. (F) Expansion of the *dei^ChO^-GFP* marker, which normally labels the cap and ligament cells, into the sensory unit was evident in larvae in which *pros*, *sens*, *raw*, *Rac1* or *Dad* were knocked down. (G) Downregulation of *Egfr*, *cpo*, *cg13653*, *ed*, *Eb1*, *pyr*, *fry*, *sr*, *mys*, *and WASp* resulted in the development of smaller CA cells that often failed to properly anchor the cap cells. (H) Downregulation of *sens*, *Rac1* and *raw* affected the cap cells on their ventral side, where they attach to the scolopale cells. (I) Abnormal elongation of the ligament cells was caused by the knockdown of *dei* and *αTub85E*. A less dramatic phenotype was caused by the loss of *mys*, *Rac1* and *sr*. Knocking down *sr* or *β1Tub* specifically in the ligament cells led to their extreme elongation. (J) Downregulation of *β1Tub*, *Tbce*, *CCT8*, *shot*, *sr* and *αTub85E* caused abnormal elongation of the attachment cells.

The screen identified 31candidate genes required for different aspects of cell fate determination, differentiation and morphogenesis of these cells (Figure 6) and provided new entry points to the study of ChO cell mechanics. One important outcome of the cell-specific differentiation programs characterizing each of the ChO cell types is the differential response of the cells to forces imposed on them by larval growth and the consequent stretching of the organ. In this respect, perhaps the most interesting group of genes identified in the screen includes genes required for the differential elongation of ChO cells. Although the phenotypic analysis of the identified genes was restricted to morphological parameters, namely the extent of cell elongation, we assume that the observed morphological alterations reflect, at least in part, changes in cell mechanics and are thus expected to affect mechanosensing. Functional studies are required to test this assumption. Interestingly, two major aberrant cell-elongation phenotypes were observed in the screen: over-elongation of the ligament cells, or over-elongation of the attachment cells, both at the expense of cap cell elongation (Figure 6). The loss of either *dei* or *αTub85E* led to extreme elongation of the ligament cells, and localized knockdown experiments pointed to the role of these genes in the development of ligament cells that do not elongate during organ stretching.

Another group of genes, consisting mainly of β-tubulins and microtubule-associated proteins (*Tbce*, *CCT8*, and *shot*) primarily affected the ability of the attachment cells to resist stretching and avoid cell elongation. Three other genes, *sr*, *mys*, and *Rac1*, affected ligament and/or CA cell elongation, in addition to affecting other aspects of ChO morphogenesis, such as the attachment between the cap and the CA cells.

The differential effect of knocking-down different tubulin genes is intriguing for two reasons. First, reducing the availability of either the *α* or the *β* tubulin monomers is expected to have a detrimental effect on the primary construction and maintenance of the microtubules. Second, the *αTub85E* and *βtub56D* (*β1Tub*) genes are similarly expressed in both the ligament cell and the attachment cells and it is not clear why each of the cell types shows higher sensitivity to the loss of one of them. Several explanations, or a combination thereof, are possible. One obvious explanation could be the availability of additional tubulin isotypes expressed within the same cells that can compensate for the loss of the specific knocked-down isotype. Differences in the expression levels of various tubulin genes together with different efficacies of the RNAi transgenes could also affect the sensitivity of the different cells to knockdown of specific tubulin isotypes. Another point to be considered is that *α* and *β* tubulin molecules differ in the post-translational modifications they go through, such as the tyrosination/detyrosination and acetylation of *α* but not *β* tubulins, which are associated with stabilized, long-lived microtubules, or as was recently suggested, render microtubules mechanically resistant to compressive forces (Xu *et al.* 2017; Janke and Montagnac 2017). It is possible that the microtubule population and, moreover, their dynamics in the attachment cells differs from that of the ligament cells thus making these cells more or less sensitive to the loss of specific *α* or *β* isotypes and their unique modifications.

Even more puzzling is the very different behavior of the cap cell during the ChO’s elongation phase. All four types of ChO accessory and attachment cells contain abundant microtubules and the cytoplasm of the cap cell, in particular, is densely packed with microtubules. Even though the cap cell expresses high levels of *dei*, *αTub85E* and *β1Tub*, which seems to protect the CA and ligament cells from stretching, this cell increases its length more than 10-folds during larval growth. Perhaps a key to the differential responses of the cap and ligament cells to stretching is the presence of the structurally divergent mesodermal variant, *β3Tub*, which is expressed in the ChO exclusively in the cap cells and only toward the end of embryogenesis shortly before larval hatching (Matthews *et al.* 1990; Kaltschmidt *et al.* 1991; Hinz *et al.* 1992; Buttgereit and Renkawitz-Pohl 1993; Dettman *et al.* 2001). It was previously suggested by (Dettman *et al.* 2001) that *β3Tub* reduces the level of cross-linking between microtubules, allowing for their sliding past each other and enabling cell elongation. Unfortunately, the one *β3Tub*–directed RNAi strain that caused a phenotype when expressed in the ChO lineage is cross-reactive with the *β1Tub* gene. Thus, it is impossible to conclude from the RNAi data about the unique role of *β3Tub* in cap cell morphogenesis. Given that the available loss-of-function alleles of *β3Tub* and *β1Tub* are lethal (Myachina *et al.* 2017), better genetic tools that allow cell-specific knockout of *β3Tub* or *β1Tub* within the ChO are required for distinguishing between the roles played by each of these tubulin isotypes. Additional tools are also required to allow for cap cell-specific knockdown of genes using RNAi transgenes. Such tools will enable us, for instance, to test whether *dei* is required in the cap cell for its ability to elongate, in addition to its role in keeping the ligament cells short, and will allow us to conduct an RNAi screen for genes that are required for cap cell elongation.
